# Synovial fluid proteome in rheumatoid arthritis

**DOI:** 10.1186/s12014-016-9113-1

**Published:** 2016-06-05

**Authors:** Mitali Bhattacharjee, Lavanya Balakrishnan, Santosh Renuse, Jayshree Advani, Renu Goel, Gajanan Sathe, T. S. Keshava Prasad, Bipin Nair, Ramesh Jois, Subramanian Shankar, Akhilesh Pandey

**Affiliations:** Institute of Bioinformatics, International Technology Park, Bangalore, 560066 India; Amrita School of Biotechnology, Amrita University, Kollam, 690525 India; Department of Biotechnology, Kuvempu University, Shankaraghatta, 577451 India; Manipal University, Madhav Nagar, Manipal, 576104 India; Department of Rheumatology, Fortis Hospital, Bangalore, 560066 India; Department of Rheumatology, Medical Division, Command Hospital (Air Force), Bangalore, 560007 India; McKusick-Nathans Institute of Genetic Medicine, Johns Hopkins University School of Medicine, 733 N. Broadway, BRB 527, Baltimore, MD 21205 USA; Department of Biological Chemistry, Johns Hopkins University School of Medicine, Baltimore, MD 21205 USA; Department of Pathology, Johns Hopkins University School of Medicine, Baltimore, MD 21205 USA; Department of Oncology, Johns Hopkins University School of Medicine, Baltimore, MD 21205 USA

**Keywords:** Lubricant, Bone repair, Neovascularisation, Hyaluronic acid, Osteoclastogenesis, Apoptosis, Angiogenesis

## Abstract

**Background:**

Rheumatoid arthritis (RA) is a chronic autoinflammatory disorder that affects small joints. Despite intense efforts, there are currently no definitive markers for early diagnosis of RA and for monitoring the progression of this disease, though some of the markers like anti CCP antibodies and anti vimentin antibodies are promising. We sought to catalogue the proteins present in the synovial fluid of patients with RA. It was done with the aim of identifying newer biomarkers, if any, that might prove promising in future.

**Methods:**

To enrich the low abundance proteins, we undertook two approaches—multiple affinity removal system (MARS14) to deplete some of the most abundant proteins and lectin affinity chromatography for enrichment of glycoproteins. The peptides were analyzed by LC–MS/MS on a high resolution Fourier transform mass spectrometer.

**Results:**

This effort was the first total profiling of the synovial fluid proteome in RA that led to identification of 956 proteins. From the list, we identified a number of functionally significant proteins including vascular cell adhesion molecule-1, S100 proteins, AXL receptor protein tyrosine kinase, macrophage colony stimulating factor (M-CSF), programmed cell death ligand 2 (PDCD1LG2), TNF receptor 2, (TNFRSF1B) and many novel proteins including hyaluronan-binding protein 2, semaphorin 4A (SEMA4D) and osteoclast stimulating factor 1. Overall, our findings illustrate the complex and dynamic nature of RA in which multiple pathways seems to be participating actively.

**Conclusions:**

The use of high resolution mass spectrometry thus, enabled identification of proteins which might be critical to the progression of RA.

**Electronic supplementary material:**

The online version of this article (doi:10.1186/s12014-016-9113-1) contains supplementary material, which is available to authorized users.

## Background

Rheumatoid arthritis (RA) is known to be a chronic and pathologically complex autoimmune disorder of joints [1, 2]. Progressive destruction of cartilage and bone over a period of 20 years can completely disable a patient diagnosed with RA [1]. The etiology and pathophysiology of this disease are not well understood and early diagnosis still remains challenging [2]. The mortality in patients mainly results from involvement of the cardiovascular system [3, 4] and renal complications [5]. The prevalence of this disease worldwide is about 1 % [6] along with a reduction in life expectancy by 3–18 years [7]. Some biomarkers that have been correlated with disease activity include the S100 proteins, matrix metalloproteinases and serum amyloid proteins [8–10]. Diagnostic markers include citrullinated proteins and anticyclic antibodies in addition to the rheumatoid factor [11–14]. The use of these markers in clinical medicine is still fraught with difficulties because of limitations on both sensitivity and specificity [2].

The biology of the disease is highly complex and manifests as a cascade of events observed during progression of the disease. Some of these include development of autoantibodies referred to as the rheumatoid factor, appearance of anti-citrullinated protein antibodies and bone and cartilage erosion, which eventually leads to systemic manifestations including involvement of the cardiovascular system and the kidneys [2, 15, 16]. The critical mediators in RA are considered to be pro-inflammatory cytokines such as RANKL, TWEAK and granulocyte macrophage colony stimulating factor (GM-CSF) [17–19]. Progressive erosion of bone in the affected sites manifests as a pseudotumoral condition of the synovium called pannus where all these complex cascades of events occur [20].

Identification of biomarkers with potential use for diagnostics and therapy is critical to make further progress in improving the clinical outcomes for patients with RA [21]. Mass spectrometry, in particular, could be useful for discovery of protein biomarkers [22]. Ideally, the underlying biological events in an RA patient are potentially reflected at the site of pathogenesis. To this end, we cataloged proteins from synovial fluid aspirated from knee joints of patients with RA using mass spectrometry-based proteomics [23]. The synovial fluid is a lubricant, composed of hyaluronic acid, inflammatory cells and proteins released from synovial fibroblasts, synovial membrane and inflammatory cells [24]. To our knowledge, there is no published study describing global profiling of the synovial fluid proteome from RA patients. However, there are multiple differential proteomic studies using synovial fluid samples of RA cases [25–27].

Similar studies describing global protein profiling in other human body fluids including urine, haemodialysis fluid, ovarian follicular fluid, pancreatic juice and bile have already been carried out by our group [28–31]. We generated a comprehensive catalog of proteins from 20 synovial fluid samples, which revealed diverse families of proteins with functions ranging from osteoclastogenesis and angiogenesis to atherogenesis. We believe that our data might provide more insights into the pathogenesis of RA and aid in developing clinical and therapeutic markers.

## Methods

### Sample preparation

Blood free synovial fluid samples from 20 RA cases were collected in Na-heparin coated BD vacutainers (cat no. 367883367883). During every collection, the sample was centrifuged at 2000 rpm for 10 min and thereafter the cell free supernatant was kept at −80 °C [32]. Sample collection was done from Fortis Hospitals, Bangalore, India and from Armed Forces Medical College, India after obtaining ethics committee clearance from the respective hospitals. Patients with RA were included who fulfilled the ACR EULAR 2010 criteria [33]. All the patients had symmetrical polyarthritis with high acute phase reactants (ESR and/or CRP). They were all positive for either rheumatoid factor or anti Cyclic citrullinated peptide (anti CCP) or both. They had mean disease duration of 37.45 months and had active disease at time of inclusion in study. Synovial fluid was collected from the knee joint in all patients. Informed consent was obtained from all the patients. The clinical profile and demographic details are included in Additional file 1: Table S1.

### Multiple lectin affinity chromatography

Glycoprotein enrichment from 20 pooled synovial fluid samples containing 2.5 mg proteins was carried out by using a mixture of three agarose-bound lectins, Wheat Germ Agglutinin, Concanavalin A and Jacalin (Vector laboratories, USA), as described previously by our group [32, 34]. Briefly, pooled lectin beads were incubated with protein samples in Tris-buffered saline (0.05 M Tris–HCl, pH 7.5, 0.15 M NaCl). After overnight incubation at 4 °C, the bound glycoproteins were eluted using competitive elution with a mixture of 100 mM of M-pyranoside, galactose, melibiose and *N*-acetyl glucoseamine in Tris-buffered saline, pH 7.5. The eluates were then washed and concentrated using 3 kDa MWCO filters (Amicon, Millipore, Ireland). The multilectin affinity approach yielded ~250 µg equivalent proteins and then stored at −20 °C until further use. These enriched proteins were then fractionated in SDS-PAGE followed by in-gel trypsin digestion.

### Multiple affinity removal system (MARS) for depleting abundant proteins

MARS-14 mini spin column (Agilent Technologies, Santa Clara, USA) was used to deplete top 14 abundant proteins (albumin, IgG, antitrypsin, IgA, transferrin, haptoglobin, fibrinogen, alpha2-macroglobulin, alpha1-acid glycoprotein, IgM, apolipoprotein AI, apolipoprotein AII, complement C3, and transthyretin) from synovial fluid. Briefly, 20 µl synovial fluid was reconstituted in 140 µl of load/wash buffer (Buffer A, Agilent Technologies, Santa Clara, USA) and the loaded MARS spin column was then centrifuged at 2000 rpm for 10 min at room temperature. The flow through was collected separately and using elution buffer (Buffer B, Agilent Technologies, Santa Clara, USA), the bound high abundant proteins were eluted out. The entire protocol was followed as per the manufacturer’s instructions. A total of 5 mg equivalent protein was depleted to 250 µg, out of which 150 µg was separated on SDS-PAGE followed by in-gel trypsin digestion. Rest of 100 µg equivalent proteins was subjected to in-solution trypsin digestion followed by strong cation exchange chromatography (SCX)-based fractionation.

### In-gel trypsin digestion

We carried out in gel-based trypsin digestion of each of the multilectin enriched proteins and MARS depleted proteins. SDS PAGE gels (10 %) were stained using colloidal-Commassie stain. Protein bands were excised and thereafter in-gel trypsin digestion was carried out, as described previously [30]. Briefly, protein bands were excised and chopped into 1 mm^3^ pieces. The gel pieces were destained in 40 % acetonitrile in 40 mM ammonium bicarbonate. Proteins were reduced using 5 mM di-thio-threitol (DTT) followed by alkylation using 10 mM iodo-acetamide (IAA). In-gel trypsin digestion was carried out at 1:20 enzyme to protein ratio overnight at 37 °C. Peptides were extracted, desalted and kept at −20 °C till further analysis.

### Strong cation exchange chromatography (SCX)

The remaining set of MARS-14 depleted proteins (~100 µg equivalent) was subjected to reduction and alkylation followed by in-solution trypsin digestion as described previously [30]. Resulting peptides were reconstituted in SCX solvent A (10 mM KH_2_PO_4_, 25 % acetonitrile pH 2.8) and loaded on a PolySULPHOETHYL A column (PolyLC, Columbia, MD, USA) using an Agilent 1200 HPLC system containing a binary pump, UV detector and a fraction collector. Peptides were fractionated by a 50 min gradient of 8–50 % solvent B (350 mM KCl in solvent A) and fractions were collected using fraction collector. The complexity of each fraction was determined based on UV absorbance at 214 nm. The fractions were then cleaned using custom made C_18_ stage-tips (3 M Empore high-performance extraction disks, St. Paul, Minnesota, USA) and were then vacuum dried and kept at −20 °C until further analysis.

### LC–MS/MS analysis

Peptide fractions were analysed on LTQ Orbitrap XL ETD mass spectrometer (Thermo, San Jose, CA, USA) interfaced with Eksigent nanoLC400 (AB SCIEX, CA, USA), to perform a reverse phase separation of peptides. At first, peptides were enriched on a trap column (75 µm X 2 cm, Magic-C_18-AQ_ material 5 µm, 100 Å) with solvent A (0.1 % formic acid) at a flow rate of 4 µl/min. The peptides were then resolved with a flow rate of 350 nl/min on a 10 cm long analytical column packed with 5 µm Magic-C_18_-AQ material (Michrom Bioresources, Inc., Auburn, CA, USA) by a gradient of 10–60 % solvent B (90 % acetonitrile in 0.1 % formic acid) over 60 min. The MS spectra were acquired in a data dependent manner in the Orbitrap at a mass resolution of 60,000 at 400 *m*/*z* while the MS/MS spectra were acquired in a linear ion trap (LTQ) mass analyzer. Nine most abundant precursor ions from a survey scan within *m*/*z* range from 350 to 1800 with a minimum signal threshold of 1000 were isolated with a 4 Da window and fragmented by CID with 35 % normalized collision energy. Dynamic exclusion was set to 90 s with a 7 ppm mass window. Maximum ion injection times were set to 10 ms for MS and 100 ms for MS/MS. The automatic gain control targets were set to 5 × 10^5^ for MS in the Orbitrap, 1 × 10^4^ for MSn in the LTQ.Xcalibur (version 2.0.7) was used for data acquisition.

### Data analysis

Protein identification was carried out using Mascot and Sequest search algorithms through the Proteome Discoverer software suite 1.3 (Thermo Scientific, Bremen, GmBH). Searches were carried against Human RefSeq protein database (Release 50, containing 33,249 protein entries). Trypsin was used as protease with maximum 1 missed cleavage allowed. Carbamidomethyl of Cysteine (C) was set as a static modification and protein N-terminal Acetylation, Oxidation of methionine (M) and deamidation of Asparagine (N) and glutamine (Q) were set as dynamic modifications. MS tolerance was set to 20 ppm while MS/MS tolerance was set to 0.8 Da. Subsequently, the identified peptides were filtered-based on false discovery rate (FDR) cut-off of 1 %. In addition to the full tryptic searches, we also carried out semi-tryptic searches using Mascot algorithm with all the previously mentioned modifications.

### Bioinformatics analysis

Gene ontology (GO)-based analysis was performed to classify proteins based on molecular function, biological process and subcellular localizations by the use of our in-built repository, Human Protein Reference Database (HPRD) (http://hprd.org) [35]. Using an in-house generated database called the Plasma Proteome Database (PPD) (http://www.plasmaproteomedatabase.org/), a publicly available repository of plasma proteins with published evidence [36], we have segregated our protein list into two sets, one set comprising proteins that were already reported in plasma with literature evidence and the other set with no published evidence.

## Results and discussion

**Fig. 1:**
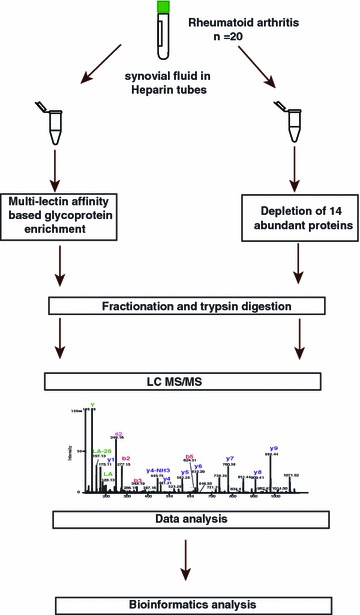
Schematic of the work flow implemented in the study. Synovial fluid samples were collected from 20 RA patients. Equal amounts of proteins were taken from all samples and pooled together followed by two sets of protein enrichment: glycoprotein enrichment using multiple lectins and depletion of abundant proteins using MARS14. The enriched proteins were thereafter taken for fractionation and trypsin digestion. The fractionated tryptic peptides were then analyzed in a high resolution mass spectrometer. The data acquired were processed and subsequently analyzed using appropriate software

Our comprehensive protein profiling approach utilized a nanoflow LC coupled with high resolution mass spectrometry. The work flow employed for this study is shown in Fig. 1. The complexity of protein composition was reduced by subjecting the 20 pooled RA synovial fluid samples to depletion and multilectin affinity-based glycoprotein enrichment. Depletion of 14 most abundant proteins was done using MARS Human 14 columns. Glycoprotein enrichment was carried out by using a mixture of three lectins—Concanavalin A, Jacalin and wheat germ agglutinin [32]. Use of multiple lectins allowed us to specifically enrich both *N*- and *O*-linked glycoproteins, thereby increasing the coverage of glycoproteins [37]. A total of 956 proteins were identified from this analysis (Additional file 2: Table S2). A partial list of previously reported and novel proteins is provided in Tables 1 and 2, respectively.

**Table 1:** A partial list of proteins identified that were previously reported

Gene symbol	Protein	Functional role in RA
*TIMP1*	Metalloproteinase inhibitor 1	MMP inhibitor
*C1QA*	Complement C1q	Immune response
*MMP9*	Matrixmetalloproteinase 9	Extracellular matrix (ECM) degradation
*S100A8*	Protein S100A8	Pannus formation
*PDL2*	Programmed death ligand 2	Inhibitor of T cell signaling
*NOTCH2*	Neurogenic locus notch homolog protein 2	Synovial hyperplasia and osteoclastogensis

**Table 2:** Partial list of novel proteins

Gene symbol	Protein	Biological role
*CDH13*	Cadherin 13	Cell adhesion
*FBLN1*	Fibulin-1	Antiangiogensis
*ITIH1*	Inter-alpha-trypsin inhibitor heavy chain H1	Hyaluronan binding
*LCN2*	Neutrophil gelatinase-associated lipocalin	Renal failure
*MTPN*	Myotrophin	Cardiac hypertrophy
*CASP14*	Caspase 14	Anti-apoptosis
*HABP2*	Hyaluronan-binding protein 2	Atherosclerosis
*OSTF1*	Osteoclast stimulating factor 1	Bone resorption

### Summary of previously reported proteins in RA

#### Inflammatory mediators

We identified a number of proteins involved in inflammation. These proteins included complement proteins, C1q, C1r, C1s, C2, C3, C4 and C5. Synovial tissues of RA patients have been reported to express almost all of the complement genes [38, 39]. Complement activation has been considered as a target for therapeutic approaches. CD93, a cell surface glycoprotein and an inflammatory mediator, was identified in our study. This molecule has already been reported to be involved in the pathogenesis of the disease [40]. It induces the differentiation of monocytes to macrophage-like cells and triggers the expression of tumor necrosis factor alpha (TNFα) [40]. We observed both CSF1 and its receptor, CSF1R, among the identified proteins. The CSF-CSFR complex is reported to augment osteoclastogenesis through proliferation, differentiation and fusion of osteoclast precursors [41]. Additionally, we identified osteopontin (OPN), another positive regulator of osteoclastogenesis coupled to the RANKL pathway [42]. Identification of tumour necrosis factor receptor 2 (TNFRSF1B), known as TNFR2, in the list of identified proteins is also in agreement with studies showing higher levels in synovial fluids of RA patients [43].

### S100 proteins

**Fig. 2:**
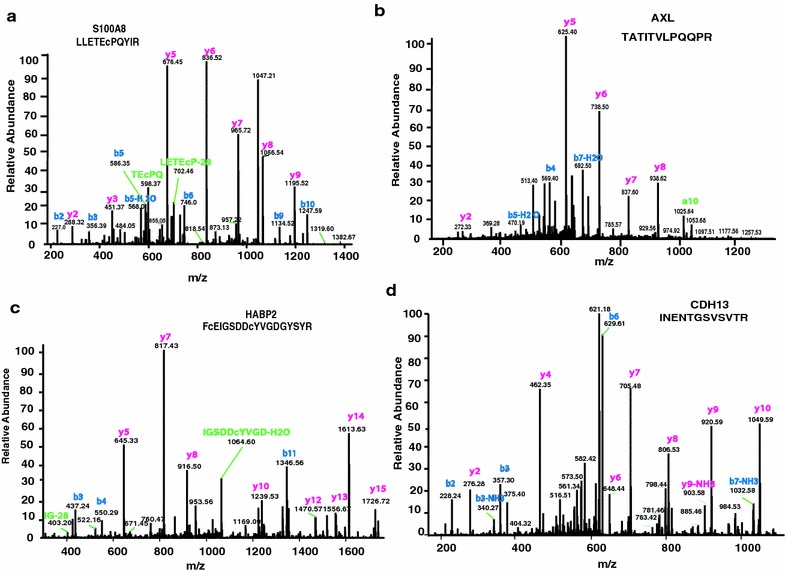
Tandem mass spectral representations of peptides with their corresponding proteins, Protein S100A8 (**a**), tyrosine-protein kinase receptor UFO, AXL (**b**), Hyaluronan Binding protein 2, HABP2 (**c**) and Cadherin 13, CDH13 (**d**). The peptide sequences have been mentioned with every corresponding protein as illustrated

The integral hallmark in RA is pannus formation, which refers to abnormal growth of blood vessels leading to a pseudotumerous condition. We identified several markers manifested in pannus formation that belong to the S100 family of proteins—notable ones include S100A11, S100A8 and S100A9 [8, 44]. Formation of the S100A8/(S100A9)_2_ heterotrimer complex referred to as calprotectin has been considered as an acute stage marker of RA [44–46]. This complex is reported to be involved in inducing inflammatory reactions in the microenvironment, it has also been found to overexpress MMPs leading to cartilage destruction in murine models of arthritis [47]. An MS/MS spectrum of one of the representative peptides of S100A8 is shown in Fig. 2a.

### Enzymes and inhibitors

We identified protein-arginine deiminase type 2 (PADI2) in our study. It belongs to the peptidyl arginine deiminase family of enzymes that carries out citrullination reactions, the post-translational deimination reaction of proteins accompanied by the conversion of arginine residues into citrullines [48]. Citrullination of proteins like plasminogen and fibrinogen is predominant in RA in which the citrullinated proteins also serve as autoantigens [49]. The critical function of this enzyme in RA has been studied previously [50]. We also observed angiotensin converting enzyme (ACE) which has been previously reported to be overexpressed in synovial stroma in patients with RA as compared to those with osteoarthritis [51]. An increase in the levels of ACE essentially reflects the production of angiogensin II, which is a local vasoconstrictor [51]. From this work, we observed matrix metalloproteinases (MMPs) and cysteine proteases, which are critical mediators of bone erosion. MMPs including the MMP1, MMP3 and MMP9 were detected in our study. These are collagenases that degrade proteins of the extracellular matrix in the affected joints [9, 52]. Cysteine proteases, referred to as the cathepsins, were also found and they include cathepsin B, D, H, S and Z types and are actively involved in bone resorption [53].

### Growth factors and binding proteins

One interesting aspect of our findings is that we identified a broad array of growth factors. Growth factors are known inducers of a number of biological events including cellular growth, proliferation and cellular differentiation. Together with cytokines they play significant roles in the microenvironment of the rheumatoid synovium [54]. Several such events are yet to be understood in the manifestation of the complex pathogenesis of the disease [54]. Connective tissue growth factor (CTGF) a secretory protein, with an integral role of coupling chondrogenesis (cartilage formation) with neo-angiogenesis, was found in our protein list [55]. It has recently been suggested as a potential therapeutic target for RA [56]. A recent report mentioned the Notch pathway dependent overexpression of vascular endothelial growth factor 3, FLT4 (VEGF3) which eventually enhances angiogenesis [57]. Interestingly, we found both neurogenic locus notch homolog protein 2 (NOTCH2) and FLT4 in the list, suggesting the imminent occurrence of an extensive angiogenesis in RA [57]. Notch2 protein (NOTCH2) has been reported to be overexpressed in synovial fibroblasts [58] and in synovium of RA patients [55]. Association of Notch signaling with osteoclastogenesis has also been considered a significant contributor to the pathogenesis of RA [59]. Its potential role of inducing the expression of MMPs in endothelial cells, through a VEGF dependent manner, provided evidence in regulating vascular endothelial cell morphogenesis [60]. Role of vascularisation in the synovium of RA patients by AXL receptor tyrosine kinase has also been studied earlier; thus its presence in our list is in agreement with previous studies [61] (MS/MS spectrum of one its peptides is shown in Fig. 2b). The presence of hepatocyte growth factor (HGF), which is mainly secreted by the mesenchymal stem cells and belongs to the plasminogen subfamily [62] was also observed. This multifunctional protein, apart from its role in angiogenesis [63], is found to be critical to osteoclastogenesis where it can substitute the role of MCSF in the presence of RANKL [64].

We observed several insulin like growth factor binding proteins, known as IGFBPs (IGFBP2, IGFBP3, IGFBP4, IGFBP5, IGFBP6 and IGFBP7) in our dataset. Regulators of cell division, the insulin-like growth factors (IGF-1 and 2), in particular, form complexes with the specific binding proteins (IGFBPs) [65]. Till date, six IGFBPs have been found and are associated with a wide range of functions. According to a previous report, they seem to activate or inhibit the functioning of IGFs, based upon the microenvironment [65]. In an earlier study in RA patients, IGFBP3 was found to inhibit cartilage synthesis by blocking the insulin growth factor1 (IGF1) mediated chondrocyte activation of cartilage synthesis [66].

### Extracellular matrix proteins

We observed several extracellular matrix proteins that were already reported in RA. Bone repair, an integral part of bone homeostasis in RA, is primarily aided by the different members of the extracellular matrix proteins. The vital members identified include collagens (COL12A1, COL14A1, COL15A1, COL18A1, COL1A1, COL1A2, COL2A1, COL3A1, COL5A1, COL6A1, COL6A2 and COL6A3), cytokeratins (KRT1, KRT10, KRT14, KRT16, KRT2, KRT5, KRT6A, KRT6B, KRT74, KRT77 and KRT9) and cartilage oligomeric matrix protein (COMP). The overexpressed cytokeratins in synovial membranes of RA patients have been already reported [67]. The higher levels of collagens in synovial fluids of RA patients have been observed to induce the expression of cytokines in mononuclear cells, thus exaggerating inflammation [68]. COMP has recently been named as a prognostic and diagnostic marker of RA [69]. We also observed lumican (LUM), a proteoglycan component which binds to collagens and its secretion locally reflects bone repair [70]. Extracellular matrix protein 1 (ECM1) is an extracellular matrix marker protein in RA. It is involved in regulating bone formation and promotes angiogenesis [71]. In our previous study, we identified the overexpression of LUM and ECM1 in RA with respect to spondyloarthropathy [32].

### Vascular cell adhesion proteins

The cell adhesion molecules (CAMs) participate in different types of homo and heterotypic cell–cell interactions, the characteristic events in RA. We identified all three major families of these proteins—L-Selectin (SELL), cadherins (CDH1, CDH5, CDH5 and CDH6), neural cell adhesion molecule 1 (NCAM1), vascular cell adhesion molecule (VCAM1) and intercellular adhesion molecule 1 (ICAM1)—all of whom have been implicated in the pathogenesis of RA [72]. VCAM1 has been found to be a fundamental player of T cell infiltration [73] and an indicative marker of endothelial dysfunction in RA [74]. An increase in the levels of the different vascular cells adhesion proteins in synovial fluid have been proposed to reflect an increase in atherosclerosis, a leading cause of death in RA patients [74–76].

### Membrane proteins

Several membrane proteins were found in our list providing evidence of the proteolytic reactions occurring in the microenvironment. Decay accelerating factor (CD55), a 70-kDa membranous protein, has been reported to be a critical co-stimulator of T cell proliferation [77] and was found to be associated with RA [78]. We have found an essential component of the T cell tolerance, PD-1 ligand, a membrane protein expressed in antigen presenting cells which binds to PD-1 in T cells that may induce T cell dysfunction [79, 80], a critical condition in RA.

### Summary of novel proteins

In this study, we identified several novel proteins in synovial fluid of RA patients. Hyaluronan-binding protein 2 (HABP2), an extracellular serine protease that has been reported to participate in negatively regulating the vascular integrity by RhoA/Rho kinase signaling pathway was identified [81]. An MS/MS spectrum of one of its peptides is shown in Fig. 2c. Angiotensin, a potent inducer of neovascularisation, was found in our list [82]. Fibulin-1 (FBLN1) is a secretory glycoprotein characterized by the presence of repeated growth factor like domains and a unique C-terminus structure [83]. This protein has been shown to have anti-angiogenic activity [83]. Neutrophil gelatinase-associated lipocalin, (LCN2) is considered as a potential biomarker of acute kidney injury [84]. It is an iron binding extracellular protein usually expressed by granulocytes and is involved in reducing angiogensis by repressing the expression of *VEGF* [85]. Cadherin 13 (CDH13), a member of the cadherin superfamily of adhesion molecules was never reported in RA. This protein mediates a calcium-dependent cell–cell adhesion in all tissue types [86] (tandem MS spectrum of one of its representative peptides is provided in Fig. 2d). Osteoclast-stimulating factor 1 (OSTF1) is an intracellular protein and has been reported to induce osteoclast formation and thus essentially increases bone resorption [87]. It acts via interactions with c-Src or other Src-related proteins [87]. A critical activator of T cell signalling, inducible co-stimulator ligand (ICOSLG) was identified in our study. Its binding to the receptor, ICOS, would potentially activate the T cell proliferation [88, 89]. Although its functional role has been studied in collagen-induced arthritis models in mice [90], its presence in synovial fluid of patients has never been detected to the best of our knowledge. Intriguingly, in addition to the T cell activators, we have identified some inhibitors as well, which might reflect the homeostatic activity within the joint. T cell activation signals can be inhibited by the immunoreceptor tyrosine-based inhibitory motifs (ITIMs)—one such protein, CD300A, was found in our list [91]. Additionally, we detected SH2 domain containing protein tyrosine phosphatase, Shp1 (PTPN6), an essential mediator of the CD300a mediated pathway [91].

### Gene ontology-based classification of proteins

**Fig. 3:**
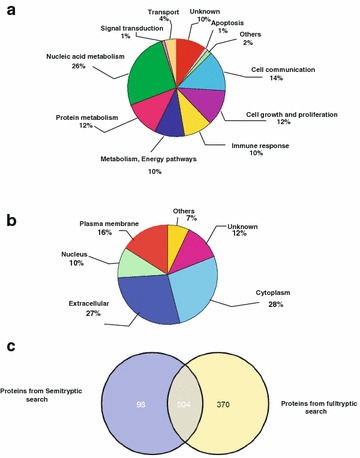
Pie chart representations of proteins in terms of biological process (**a**) and cellular component (**b**) Majority of the proteins were found to be extracellular, cytoplasmic ortransmembrane in nature. They are associated with a number of biological functions includingimmune response, metabolic reactions, cell communications and several others. A Venn diagram illustrating distribution of proteins identified from semi and fulltryptic searches used in the study (**c**). From a total of 956 proteins, 98 were derived from only the Mascot-based semitryptic searches and 504 were obtained from both the full tryptic and semitryptic searches and the rest were reported by only full tryptic constraints

To obtain a deeper biological insight into the protein list that we have generated, we classified proteins into gene ontology-based categories including molecular function, biological process and subcellular localization using HPRD (http://www.hprd.org/) (for details, please see Fig. 3a, b and Additional file 3: Table S3). We observed that the proteins are mostly either extracellular or cytoplasmic in nature, and are primarily involved in a cascade of functions, including immune response, cell growth and cell–cell communication.

### Semi-tryptic peptides

Considering the extensive proteolytic events in the pathological site of RA, we sought to identify proteins by semi-tryptic searches in addition to the fullytryptic searches. As explained earlier, in vivo derived proteolytic fragments might be identified from semi-tryptic searches [92]. Thus, we implemented this strategy to identify additional proteins by using semi-tryptic search constraints. Out of the 956 proteins identified, a total of 98 proteins were identified uniquely from the Mascot-based semi-tryptic search. From total 5884 peptides, 1831 peptides were identified by full tryptic searches while 1270 peptides were identified by semi-tryptic searches and 2783 peptides were found to be identified by both search types. The protein distribution was listed in Additional file 2: Table S2 and Fig. 3c.

### Comparison of synovial fluid proteins with plasma proteins

We overlapped our data with the protein list from PPD (http://www.plasmaproteomedatabase.org/), a repository of proteins reported in plasma/serum with published literature evidence [93]. There were 130 proteins found to be uniquely present in synovial fluid and till date they were not observed in circulation as reported in the serum/plasma (see Additional file 3: Table S3). Interestingly, in this protein list, we found a novel cytokine involved in osteoclastogenesis, secreted osteoclastogenic factor of activated T cells (SOFAT). This molecule has been reported to be a novel cytokine which induces the expression of IL-6 in osteoblasts. Interleukin-6 is a cytokine that upregulates osteoclastogensis in RA in a RANKL-independent manner [94]. An integral protein observed from the list is phosphatidylinositide phosphatase SAC2 (INPP5F), which contains a Sac domain and has been implicated in cardiovascular disorders [95]. One of its family members, inositol polyphosphate-5-phosphatase (INPP5E), a 72 kDa protein, was identified in the circulating mononuclear cells of RA patients [96]. We observed semaphorin 4A (SEMA4A), a class IV transmembrane protein that binds to the T cell immunoglobulin and mucin domain-containing protein 2 (TIM2), in activated T cells. This Sema4A–Tim2 complex triggers T cell activation [97]. Taken together, our data validates the significance of studying proximal fluids for biomarker discovery.

## Conclusions

The main objective of the study was to identify proteins that are specifically present in the pathological site called synovial fluids using high resolution MS. This way it would give a better picture to understand the biology of RA.

From the list, we observed proteins that are critical players of a cascade of biological events including angiogenesis (AXL, FLT4, Notch2), osteoclastogenesis (CSF/CSFR, NOTCH2, OPN), pannus formation (S100 family), endothelial dysfunction and cell migration (ICAM, VCAM-1), activators and inhibitors of T cell signalling (CD300a, PTPN6, PDL2) and finally inflammation (TNFR2). We believe our data could be a useful resource for biomarker discovery in RA. Recently, our group has identified a number of proteins overexpressed in RA with respect to spondyloarthropathy using high resolution MS technology [32].

As the aspiration of joint fluids from normal individuals cannot be carried out for ethical reasons and because there is very little synovial fluid in the absence of any inflammation of the joint, we could not compare RA synovial fluid proteome with that obtained from normal individuals [98]. Taken together, we propose that MS-based studies on diseased biological fluid samples would be beneficial for discovery of disease biomarkers. Our data should be valuable in understanding the role of the local milieu in the pathogenesis of RA.

## Declarations

### Data availability

The processed data is available online in Human Proteinpedia (http://www.humanproteinpedia.org/data_display?exp_id=00852) [99], the in-house developed repository of proteins identified from mass spectrometry-based reports. The mass spectrometry proteomics data have been deposited to the ProteomeXchange Consortium (http://www.proteomexchange.org) via the PRIDE partner repository [100] with the dataset identifier PXD000740.

Following are the details:

- Project accession: PXD000740
- Reviewer account:
- Username: reviewer70321@ebi.ac.uk
- Password: OSZmk0hJ

## Additional files

10.1186/s12014-016-9113-1 Clinical details of patients used in the study.
10.1186/s12014-016-9113-1 List of proteins and their corresponding peptides identified in the study.
10.1186/s12014-016-9113-1 Details of proteins identified in the study with reports on whether or not reported in PPD and gene ontology-based classification.
