# Supplementary material for: Synovial fluid proteome in rheumatoid arthritis
Source: Clin Proteomics. 2016 Jun 5;13:12. doi: 10.1186/s12014-016-9113-1 (PMC4893419; doi:10.1186/s12014-016-9113-1)
Supplement: Supplementary file 1 — 10.1186/s12014-016-9113-1 Clinical details of patients used in the study. [file 12014_2016_9113_MOESM1_ESM.docx]

**Table S1**: Clinical profile of patients of RA used in this study

| **Number of patients** | 20 | |
| --- | --- | --- |
| **Male: Female** | 6:14 | |
| **Age group (in years)** | 40+ 5 years | |
| **Erosions on radiographs** | 14 | |
| **Disease duration (in months)**  Mean + SD  Range | 37.45 + 15.74 months | |
|  | 12-72 months | |
| **Autoantibody profile*** | **RF Positive** | **RF Negative** |
| **Anti CCP positive** | 15 | 3** |
| **Anti CCP negative** | 02 | 0 |
| **Raised acute phase reactants (ESR or CRP)** | 20 | |

*All patients were positive for RF or anti CCP or both

**All RF negative patients were positive for anti CCP
